# Investigation on Thermally Radiative Mixed Convective Flow of Carbon Nanotubes/*Al*_2_*O*_3_ Nanofluid in Water Past a Stretching Plate with Joule Heating and Viscous Dissipation

**DOI:** 10.3390/mi13091424

**Published:** 2022-08-29

**Authors:** R. Prabakaran, S. Eswaramoorthi, Karuppusamy Loganathan, Ioannis E. Sarris

**Affiliations:** 1 Department of Mathematics, Coimbatore Institute of Technology, Coimbatore 641014, Tamil Nadu, India; 2 Department of Mathematics, Dr. N.G.P. Arts and Science College, Coimbatore 641048, Tamil Nadu, India; 3 Department of Mathematics and Statistics, Manipal University Jaipur, Jaipur 303007, Rajasthan, India; 4 Research and Development Wing, Live4Research, Tiruppur 638106, Tamil Nadu, India; 5 Department of Mechanical Engineering, University of West Attica, 12244 Athens, Greece

**Keywords:** SWCNTs and MWCNTs, HAM, radiation, joule heating, viscous dissipation, suction/injection

## Abstract

The nature of this prevailing inquisition is to scrutinize the repercussion of MHD mixed convective flow of CNTs/$Al_{2}O_{3}$ nanofluid in water past a heated stretchy plate with injection/suction, heat consumption and radiation. The Joule heating and viscous dissipation are included in our investigation. The Navier–Stokes equations are implemented to frame the governing flow expressions. These flow expressions are non-dimensioned by employing suitable transformations. The converted flow expressions are computed numerically by applying the MATLAB bvp4c procedure and analytically by the HAM scheme. The impacts of relevant flow factors on fluid velocity, fluid temperature, skin friction coefficient, and local Nusselt number are illustrated via graphs, tables and charts. It is unequivocally shown that the fluid speed declines when escalating the size of the magnetic field parameter; however, it is enhanced by strengthening the Richardson number. The fluid warmness shows a rising pattern when enriching the Biot number and heat consumption/generation parameter. The findings conclusively demonstrate that the surface drag force improves for a larger scale of Richardson number and is suppressed when heightening the unsteady parameter. In addition, it is evident from the outcomes that the heat transfer gradient decreases to increase the quantity of the Eckert number in the convective heating case; however, the opposite nature is obtained in the convective cooling case. Our numerical results are novel, unique and applied in microfluid devices such as micro-instruments, sleeve electrodes, nerve growth electrodes, etc.

## 1. Introduction

The fluid thermal conductivity has been used in many scientific and technical sectors, such as microelectronics, transportation, atomic reactors, heat exchangers, cancer therapy, etc. The ordinary base fluids such as water, oils, ethylene glycol and kerosene have a smaller heat transfer phenomenon because of their weaker thermal conductivity. One of the facile ways to escalate the fluid thermal conductivity is to admix the nanoscale (1–100 nm) particles named nanoparticles into the ordinary base fluids to improve their conductivity. Imtiaz et al. [1] proved that the flow speed is enriched for larger values of the shape parameter and NPVF for the 3D flow of CNTs with the CCHF model. The problem of stagnation point flow of CNTs past a cylinder was analytically solved by Hayat et al. [2]. It has been proved that the NPVF leads to the development of the skin friction coefficient. Yacob et al. [3] noticed that the larger size of NPVF generates more heat inside the boundary for the problem of rotating flow of CNTs on a shrinking/stretching surface. The flow of water/kerosene-based CNTs over a moving plate with suction was examined by Anuar et al. [4]. Their findings unambiguously demonstrate that the SWCNTs have a more significant skin friction coefficient, and MWCNTs have a more considerable heat transfer gradient. Haq et al. [5] found that the SWCNTs have a bigger heat transfer gradient than the MWCNTs for the problem of the MHD pulsatile flow of CNTs. They consider engine oil as a base fluid.

Electrically conducting fluids play a vital role in nuclear power plants, MHD generators, plasma propulsion in astronautics, geophysics, power plants, astronomy, etc. The steady, MHD and stagnation flow of CNTs past a shrinking/stretching sheet was examined by Anuar et al. [6]. Their findings clearly demonstrate that the magnetic field parameter leads to an enhancement of the surface shear stress. Manjunatha et al. [7] examined the MHD flow of water-based CNTs on a rotating disk. It is noted that the fluid thermal profile develops when enhancing the magnetic field parameter. Acharya et al. [8] noticed a larger magnetic field parameter drop-off of the fluid heat transfer gradient in their study of the MHD flow of CNTs past a deformable sheet. The time-dependent free convective flow of Casson nanofluid past a moving plate with the impact of a magnetic field was discussed by Noranuar et al. [9]. It is evident from the outcomes that the skin friction coefficient improves when strengthening the magnetic field. The natural convective flow of MHD water-based CNTs was examined by Benos et al. [10]. Mabood et al. [11] noticed that the entropy production reduces via a magnetic field in their study of the MHD flow of Jeffrey nanofluid past a SS.

However, in the majority of the aforementioned investigations, the impact of thermal radiation on flow and heat transmission has not been considered. However, when technical procedures are carried out at high temperatures, they become significant and cannot be disregarded. The fluid flow with radiation is noteworthy in many engineering processes that occur at high temperatures in industrial processes. The heat transfer via radiation is essential in producing reliable equipment, nuclear plants, gas turbines, satellites, aircraft, missiles, spacecraft, etc. Mahabaleshwar et al. [12], in their study of the radiative flow of water-based CNTs past a stretching surface, reported that a higher radiation parameter develops a temperature gradient. The radiative flow of CNTs on a stretching sheet with a magnetic field was investigated by Shah et al. [13]. Their results unequivocally show that the radiation parameter improves the fluid warmness. Aman et al. [14] noted from the obtained results that the temperature of SWCNTs is larger than the MWCNTs for the problem of MHD water/kerosene-oil/engine-oil-based CNTs past a vertical channel. MHD mixed convective flow of CNTs on a cone with the convective heating condition was scrutinized by Sreedevi et al. [15]. Their results undoubtedly show that the skin friction coefficient develops when improving the radiation parameter. Reddy and Sreedevi [16] proved that the radiation parameter enhances the heat transfer rate in their analysis of the thermally radiative flow of CNTs inside a square chamber. The impact of radiative and unsteady MHD flow of CNTs with thermal stratification was addressed by Ramzan et al. [17]. It is noticed from their outcomes that the local Nusselt number escalates with a larger radiation parameter. Mahabaleshwar et al. ([18,19]) addressed the radiative flow of Walters’ liquid-B and couple stress fluid past an SS.

Moreover, the significance of heat generation/absorption has received considerable attention from many researchers due to its practical usage of debris, heat nuclear reactors, underground disposal of radioactive waste material and semiconductor wafers. Kataria et al. [20] detected that the heat generation/absorption parameter develops the fluid warmness in their analysis of the MHD flow of Casson fluid over an exponentially accelerated plate with heat generation/absorption. The 2D radiative flow of water-based CNTs past curved surfaces with internal heat generation was deliberated by Saba et al. [21]. It is acknowledged that the local heat flux rate declines when improving the heat generation parameter. Ojemeri and Hamza [22] unambiguously demonstrated that a higher heat source parameter improves fluid flow inside the boundary for the problem of chemically reacting MHD flow due to the effect of heat generation/absorption. Entropy optimization of MHD mixed convective flow of Cu nanofluid with a heat sink/source was portrayed by Chamkha et al. [23]. Khan and Alzahrani [24] investigated the consequences of Darcy–Forchheimer flow of nanofluid with the impact of radiation and heat generation/absorption. MHD free convective flow in a concentric annulus with heat generation/absorption was inspected by Gambo and Gambo [25]. Their results do not doubt that the changes in the heat generation/absorption parameter improve the fluid temperature.

This research aims to simulate the mixed convective flow of CNTs past a stretching plate inserted in a porous medium.

The innovation of the present exploration is:

- To investigate the MHD flow over a stretchy plate inserted in a porous medium.
- The impacts of Joule heating, viscous dissipation and radiation are also added to the heat expression.
- These types of modeled problems are used in the thermal industry for designing equipment, such as the design of electric ovens, electric heaters, microelectronics, wind generators, etc.

## 2. Mathematical Formulation

**Figure 1:**
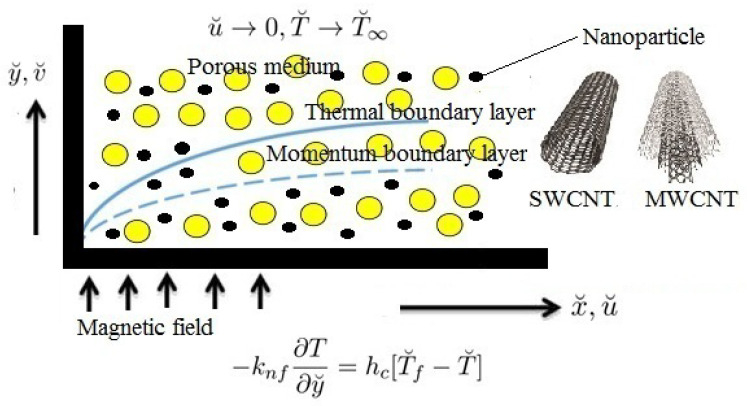
Physical configuration of the flow model.

The following flow hypothesis is used to simulate the fluid’s flow:

- The time-dependent, 2D, incompressible, electrically conducting flow of CNTs past a stretchy plate is embedded in a porous medium.
- Let the $\overset{˘}{x}$-coordinate be delineated in the plate, the $\overset{˘}{y}$-coordinate is normal to it, and the flow occurs when $\overset{˘}{y}>0$.
- The surface of the plate has a constant temperature ${\overset{˘}{T}}_{w}$, which is bigger than the ambient fluid temperature ${\overset{˘}{T}}_{\infty }$.
- The fixed magnetic field of quantity *B* is employed in the $\overset{˘}{y}$-coordinate; see Figure 1.
- The induced magnetic field is omitted because of the small size of the Reynold’s number.
- The availability of heat consumption/generation, Joule heating, and radiation impacts are included to analyze the variations of velocity, temperature, SFC and LNN.
- The characteristics of fluids are regarded as constants.

Under the above considerations, the flow model can be expressed as (see Soomro et al. [26] and Haq et al. [27])

(1) $$\begin{array}{ccc} & & \frac{\partial \overset{˘}{u}}{\partial \overset{˘}{x}}+\frac{\partial \overset{˘}{v}}{\partial \overset{˘}{y}}=0 \end{array}$$

(2) $$\begin{array}{ccc} & & \frac{\partial \overset{˘}{u}}{\partial \overset{˘}{t}}+\overset{˘}{u}\frac{\partial \overset{˘}{u}}{\partial \overset{˘}{x}}+\overset{˘}{v}\frac{\partial \overset{˘}{u}}{\partial \overset{˘}{y}}=\nu_{nf}\frac{\partial^{2}\overset{˘}{u}}{\partial {\overset{˘}{y}}^{2}}-\frac{\nu_{nf}}{k_{1}^{*}}\overset{˘}{u}-\frac{\sigma_{nf}}{\rho_{nf}}B^{2}\overset{˘}{u}+\frac{{\left(\rho \beta \right)}_{nf}}{\rho_{nf}}g\left(\overset{˘}{T}-\overset{˘}{T_{\infty }}\right)\\ & & \frac{\partial \overset{˘}{T}}{\partial \overset{˘}{t}}+\overset{˘}{u}\frac{\partial \overset{˘}{T}}{\partial \overset{˘}{x}}+\overset{˘}{v}\frac{\partial \overset{˘}{T}}{\partial \overset{˘}{y}}=\alpha_{nf}\frac{\partial^{2}\overset{˘}{T}}{\partial {\overset{˘}{y}}^{2}}+\frac{16\sigma^{*}{\overset{˘}{T_{\infty }}}^{3}}{3k^{*}{\left(\rho c_{p}\right)}_{nf}}\frac{\partial^{2}\overset{˘}{T}}{\partial {\overset{˘}{y}}^{2}} \end{array}$$

(3) $$\begin{array}{ccc} & & \;\;+\frac{Q}{{\left(\rho c_{p}\right)}_{nf}}\left(\overset{˘}{T}-{\overset{˘}{T}}_{\infty }\right)+\frac{\mu_{nf}}{{\left(\rho c_{p}\right)}_{nf}}{\left(\frac{\partial \overset{˘}{u}}{\partial \overset{˘}{y}}\right)}^{2}+\frac{\sigma_{nf}}{{\left(\rho c_{p}\right)}_{nf}}B^{2}{\overset{˘}{u}}^{2} \end{array}$$

with the boundary conditions

(4) $$\begin{array}{c} \overset{˘}{u}=\overset{˘}{U_{w}}=\frac{a\overset{˘}{x}}{1-\xi t};\;\;\;\;\overset{˘}{v}=-\frac{\overset{˘}{\nu_{0}}}{\sqrt{1-\xi t}}\\ -k_{nf}\frac{\partial \overset{˘}{T}}{\partial \overset{˘}{y}}=h_{c}\left[{\overset{˘}{T}}_{f}-\overset{˘}{T}\right]\;\;at\;\;\overset{˘}{y}=0\\ \overset{˘}{u}\to 0,\overset{˘}{T}\to {\overset{˘}{T}}_{\infty }\;\;as\;\;\overset{˘}{y}\to \infty \end{array}$$

All the symbols are shown in the nomenclature section.

Define the variables (see Upadhya et al. [28]),

(5) $$\begin{array}{c} \Upsilon =\sqrt{\frac{a}{\nu_{f}\left(1-\xi \overset{˘}{t}\right)}}\overset{˘}{y};\;\;\overset{˘}{u}=\frac{a}{1-\xi t}\overset{˘}{x}F^{\prime }\left(\Upsilon \right);\overset{˘}{v}=-\sqrt{\frac{a\nu_{f}}{1-\xi t}}F\left(\Upsilon \right);\;\;\Theta =\frac{\overset{˘}{T}-{\overset{˘}{T}}_{\infty }}{{\overset{˘}{T}}_{f}-{\overset{˘}{T}}_{\infty }} \end{array}$$

Substituting Equation (5) in Equations (2) and (3), we have

$$\begin{array}{ccc} & & A_{1}A_{2}F^{\prime \prime \prime }\left(\Upsilon \right)+F\left(\Upsilon \right)F^{\prime \prime }\left(\Upsilon \right)-{F^{\prime }}^{2}\left(\Upsilon \right)-A\left[F^{\prime }\left(\Upsilon \right)+\frac{\eta }{2}F^{\prime \prime }\left(\Upsilon \right)\right]-A_{1}A_{2}\lambda F^{\prime }\left(\Upsilon \right) \end{array}$$

(6) $$\begin{array}{ccc} & & \;\;-A_{2}A_{3}MF^{\prime }\left(\Upsilon \right)+A_{2}A_{4}Ri\Theta \left(\Upsilon \right)=0\\ & & A_{5}A_{6}\frac{1}{Pr}\Theta^{{}^{\prime \prime }}\left(\Upsilon \right)+F\left(\Upsilon \right)\Theta^{{}^{\prime }}\left(\Upsilon \right)-F^{\prime }\left(\Upsilon \right)\Theta \left(\Upsilon \right)-A\left[\Theta \left(\Upsilon \right)+\frac{\eta }{2}\Theta^{\prime }\left(\Upsilon \right)\right]+\frac{A_{6}}{Pr}\frac{4}{3}Rd\Theta^{{}^{\prime \prime }}\left(\Upsilon \right) \end{array}$$

(7) $$\begin{array}{ccc} & & \;\;+A_{1}A_{6}EcF^{\prime \prime }{}^{2}\left(\Upsilon \right)+A_{3}A_{6}MEcF^{\prime }{}^{2}\left(\Upsilon \right)+A_{6}Hg\Theta \left(\Upsilon \right)=0 \end{array}$$

with the conditions,

(8) $$\begin{array}{c} F\left(0\right)=fw,\;\;\;F^{\prime }\left(0\right)=1,\;\;\;F^{\prime }\left(\infty \right)=0\\ \Theta^{\prime }\left(0\right)=-\frac{Bi}{A_{5}}\left[1-\Theta (0)\right];\;\;\;\Theta \left(\infty \right)=0 \end{array}$$

All the parameters are shown in the nomenclature part, and the notations are defined in Appendix A.

The skin friction coefficient and local Nusselt number are expressed as follows,

$$\begin{array}{c} \frac{1}{2}Cf\sqrt{Re}=A_{1}F^{\prime \prime }\left(0\right);\;\;\;\frac{Nu}{\sqrt{Re}}=-\left[A_{5}+\frac{4}{3}Rd\right]\Theta^{\prime }\left(0\right) \end{array}$$

## 3. Solutions

### 3.1. Numerical Solutions

**Figure 2:**
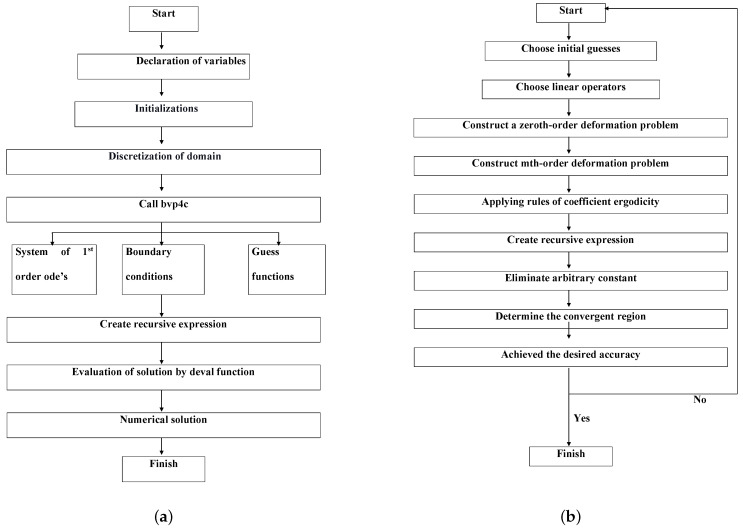
The flow chart of bvp4c (**a**) and HAM (**b**).

The amended expressions (6) and (7) and their correlated constraints (8) are numerically computed by applying the MATLAB BVP4C theory (Figure 2a) (see Eswaramoorthi et al. [29]). In this regard, initially, we convert the high-order ODEs into first-order ODEs.

Let $f=D_{1},\;f^{\prime }=D_{2},\;f^{\prime \prime }=D_{3},\;\Theta =D_{4},\;\Theta^{\prime }=D_{5}$.

The system of equations is

$$\begin{array}{ccc} & & D_{1}^{\prime }=D_{2}\\ & & D_{2}^{\prime }=D_{3}\\ & & D_{3}^{\prime }=\frac{D_{2}^{2}-D_{1}D_{3}+A\left[D_{2}+\frac{\eta }{2}D_{3}\right]+A_{1}A_{2}\lambda D_{2}+A_{2}A_{3}MD_{3}-A_{2}A_{4}RiD_{4}}{A_{1}A_{2}}\\ & & D_{4}^{\prime }=D_{5}\\ & & D_{5}^{\prime }=\frac{D_{2}D_{4}-D_{1}D_{5}+A\left[D_{4}+\frac{\eta }{2}D_{5}\right]-A_{1}A_{6}EcD_{3}^{2}-A_{3}A_{6}MEcD_{2}^{2}-A_{6}HgD_{4}}{\frac{A_{6}}{Pr}\left[A_{5}+\frac{4}{3}Rd\right]} \end{array}$$

with the corresponding conditions

$$\begin{array}{ccc} & & D_{1}\left(0\right)=fw;\;\;D_{2}\left(0\right)=1;\;\;D_{2}\left(\infty \right)=0;\;\;D_{5}\left(0\right)=-\frac{B_{i}}{A_{5}}\left(1-D_{4}\left(0\right)\right);\;\;D_{4}\left(\infty \right)=0 \end{array}$$

### 3.2. Analytical Solutions

The amended expressions (6) and (7) and their correlated constraints (8) are analytically computed by applying the HAM scheme (Figure 2b). This method was developed by Shijun Liao in 1992, and it is a powerful mathematical method for solving highly non-linear problems (see [30,31]).

Initial approximations:

$$\begin{array}{c} F_{0}\left(\Upsilon \right)=fw+\left[1-\frac{1}{e^{\Upsilon }}\right];\;\;\Theta_{0}\left(\Upsilon \right)=\frac{Bi}{\left[Bi+A_{5}\right]e^{\Upsilon }} \end{array}$$

Linear operators:

$$\begin{array}{c} L_{F}=F^{\prime \prime \prime }-F^{\prime };\;\;L_{\Theta }=\Theta^{\prime \prime }-\Theta \end{array}$$

Linear properties:

$$\begin{array}{c} L_{F}\left[\Omega_{1}+\Omega_{2}e^{\Upsilon }+\Omega_{3}\frac{1}{e^{\Upsilon }}\right]=0=L_{\Theta }\left[\Omega_{4}e^{\Upsilon }+\Omega_{5}\frac{1}{e^{\Upsilon }}\right] \end{array}$$

where $\Omega_{j};j$ = 1–5 are constants.

Zeroth-order deformation problems:

$$\begin{array}{ccc} \left(1-p\right)L_{F}\left[F\left(\Upsilon ,p\right)-F_{0}\left(\Upsilon \right)\right] & = & ph_{F}N_{1}\left[F\left(\Upsilon ,p\right),\Theta \left(\Upsilon ,p\right)\right]\\ \left(1-p\right)L_{\Theta }\left[\Theta \left(\Upsilon ,p\right)-\Theta_{0}\left(\Upsilon \right)\right] & = & ph_{\theta }N_{2}\left[F\left(\Upsilon ,p\right),\Theta \left(\Upsilon ,p\right)\right] \end{array}$$

Here p∈[0,1] is an embedding parameter, and $N_{1}$ and $N_{2}$ are non-linear operators.

The *n*th order problems:

$$\begin{array}{c} F_{n}\left(\Upsilon \right)=F_{n}^{*}\left(\Upsilon \right)+\Omega_{1}+\Omega_{2}e^{\Upsilon }+\Omega_{3}\frac{1}{e^{\Upsilon }};\;\;\Theta_{n}\left(\Upsilon \right)=\Theta_{n}^{*}\left(\Upsilon \right)+\Omega_{4}e^{\Upsilon }+\Omega_{5}\frac{1}{e^{\Upsilon }}\;\; \end{array}$$

Here $F_{n}^{*}\left(\Upsilon \right)$ and $\Theta_{n}^{*}\left(\Upsilon \right)$ are the particular solutions.

**Figure 3:**
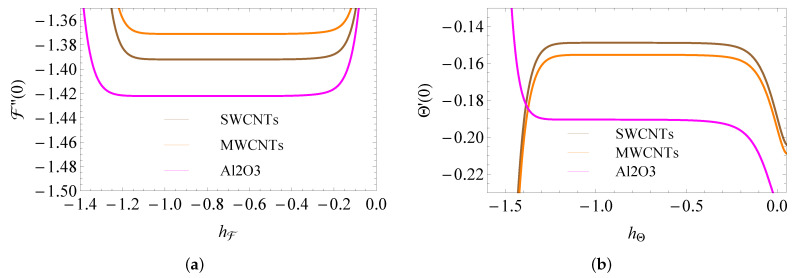
h-curves of $F^{\prime \prime }\left(0\right)$ (**a**) and $\Theta^{\prime }\left(0\right)$ (**b**).

The HAM parameters ($h_{F}$ and $h_{\Theta }$) are responsible for the solution convergency (see Loganathan et al. [32] and Eswaramoorthi et al. [33]). The limits of $h_{F}$ are [−1.15, −0.3] (SWCNTs), [−1.1, −0.35] (MWCNTs), [−1.2, −0.38] ($Al_{2}O_{3}$) and $h_{\Theta }$ is [−1.3, −0.3] (SWCNTs), [−1.25, −0.35] (MWCNTs), [−1.3, −0.4] ($Al_{2}O_{3}$) ( see Figure 3a,b).

## 4. Results and Discussion

The main objective of this segment is to show how the different pertinent flow parameters affect the fluctuations in the fluid velocity, fluid temperature, skin friction coefficient, and local Nusselt number for SWCNTs, MWCNTs and $Al_{2}O_{3}$ nanofluid. The physical properties of SWCNTs, MWCNTs, $Al_{2}O_{3}$ and water are presented in Table 1. Table 2, Table 3 and Table 4 clearly display the HAM order of approximations and numerical value for all cases. It can be noted from these tables that the 18th order is sufficient for all computations in all cases. The SFC for various values of *A*, *M*, λ, Ri, fw, Rd, Ec and Hg is shown in Table 5. This table plainly demonstrates that the plate surface drag force decreases when increasing the size of *A*, *M*, λ and fw. On the other hand, during development, it enhances the quantity of Ri, Rd, Ec and Hg. In addition, MWCNTs have more surface drag force than the SWCNTs and $Al_{2}O_{3}$ nanofluid. Table 6 shows the consequences of *A*, *M*, λ, Ri, fw, Rd, Ec and Hg on LNN. It can be observed from this table that the HTG grows at growing the quantity of *A*, Ri, fw, Rd. However, it diminishes when the values of *M*, λ, Ec and Hg are magnified. Additionally, the SWCNTs have less HTG than the MWCNTs and $Al_{2}O_{3}$ nanofluid.

**Figure 4:**
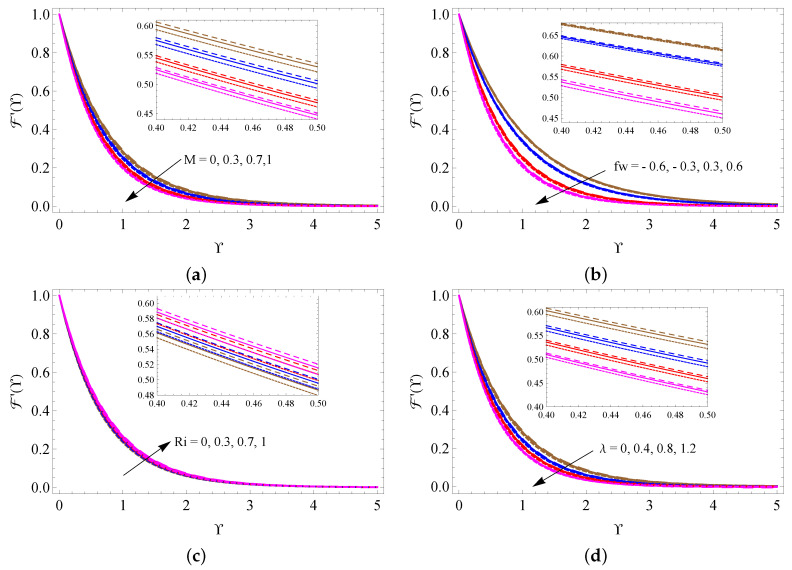
The contrast of $F^{\prime }\left(\Upsilon \right)$ against *M* (**a**), fw (**b**), Ri (**c**) and λ (**d**) for SWCNTs (solid line), MWCNTs (dashed line) and $Al_{2}O_{3}$ nanofluid (dotted line).

**Figure 5:**
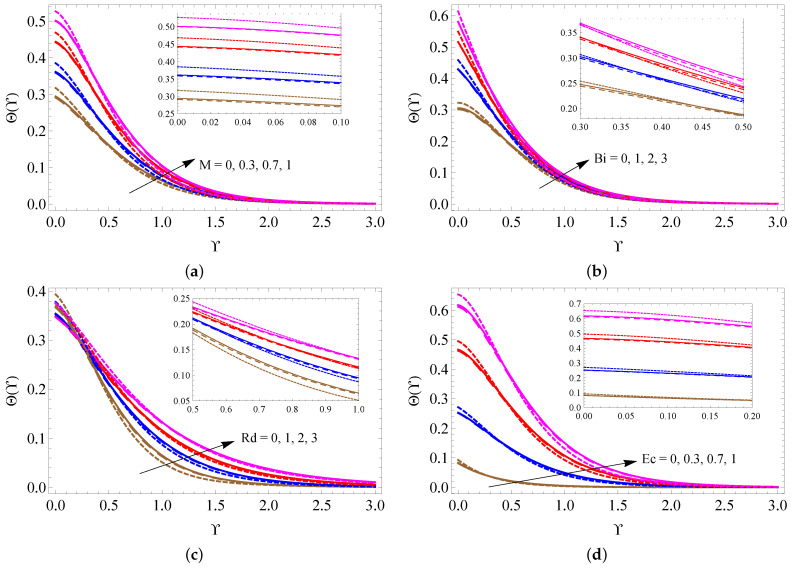
The contrast of Θ(Υ) against *M* (**a**), Bi (**b**), Rd (**c**) and Ec (**d**) for SWCNTs (solid line), MWCNTs (dashed line) and $Al_{2}O_{3}$ nanofluid (dotted line).

**Figure 6:**
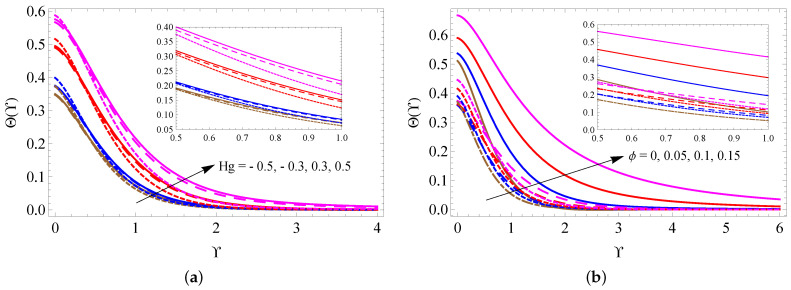
The contrast of Θ(Υ) against Hg (**a**) and ϕ (**b**) for SWCNTs (solid line), MWCNTs (dashed line) and $Al_{2}O_{3}$ nanofluid (dotted line).

Figure 4a–d is drawn to examine the alterations of *M*, fw, Ri and λ on the fluid velocity distribution. It is detected from these figures that the larger measure of Ri improves the fluid motion inside the boundary. On the contrary, the larger size of *M*, fw and λ reduces the fluid motion. The changes in fluid temperature for distinct quantities of *M*, Bi, Rd and Ec are pictured in Figure 5a–d. These figures noticeably point out that the fluid temperature increases when the values of *M*, Bi, Rd and Ec increase. Figure 6a,b is taken to analyze the change of Hg and ϕ on fluid temperature distribution. It is found from these figures that the fluid temperature escalates when raising the values of Hg and ϕ.

**Figure 7:**
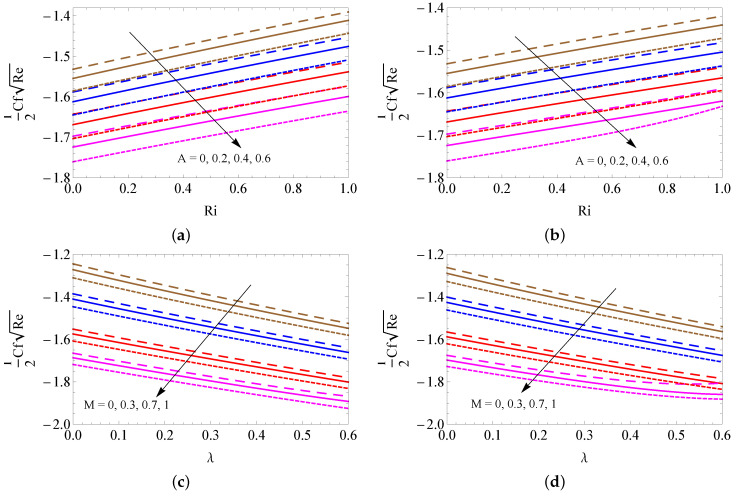
The contrast of the skin friction coefficient for different combinations of *A* and Ri (**a**,**b**) and *M* and λ (**c**,**d**) with convective heating (**a**,**c**) and convective cooling (**b**,**d**) cases for SWCNTs (solid line), MWCNTs (dashed line) and $Al_{2}O_{3}$ nanofluid (dotted line).

**Figure 8:**
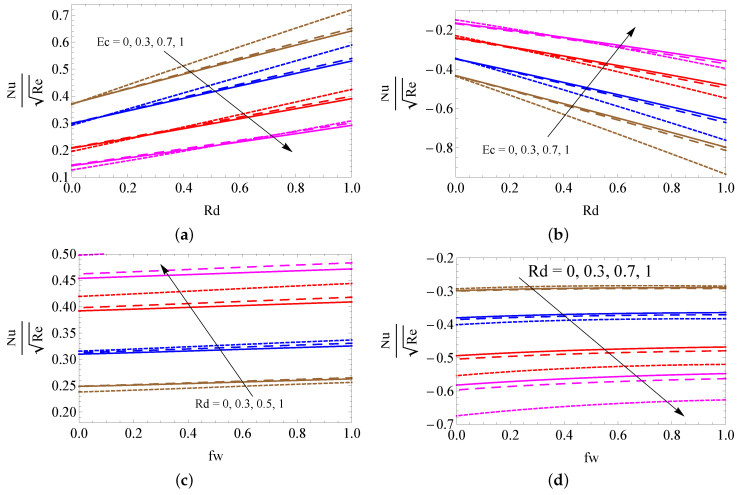
The contrast of LNN for different combinations of Ec and Rd (**a**,**b**) and Rd and fw (**c**,**d**) with convective heating (**a**,**c**) and convective cooling (**b**,**d**) cases for SWCNTs (solid line), MWCNTs (dashed line) and $Al_{2}O_{3}$ nanofluid (dotted line).

**Figure 9:**
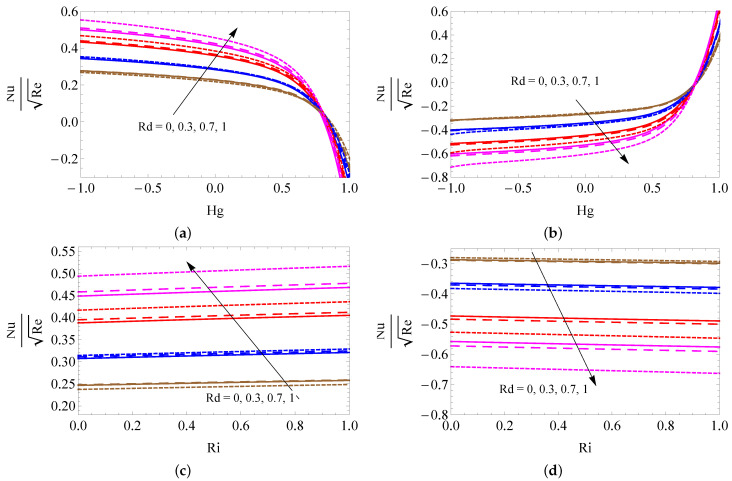
The contrast of LNN for different combinations of Rd and Hg (**a**,**b**) and Rd and Ri (**c**,**d**) with convective heating (**a**,**c**) and convective cooling (**b**,**d**) cases for SWCNTs (solid line), MWCNTs (dashed line) and $Al_{2}O_{3}$ nanofluid (dotted line).

The contrast of the skin friction coefficient for different combinations of *A* and Ri (a–b) and *M* and λ (c–d) with convective heating (a,c) and convective cooling (b,d) cases are illustrated in Figure 7a–d. A large amount of unsteady magnetic field and porosity parameters is perceived to lead to a fall out of the SFC. However, it improves when developing the Richardson number for both cases. Figure 8a–d is to used to discuss the contrast of LNN for different combinations of Ec and Rd (a–b) and Rd and fw (c–d) with convective heating (a,c) and convective cooling (b,d) cases. It is detected from these figures that the larger magnitudes of Rd and fw upsurge the heat transfer gradient, and the Eckert number weakens the LNN for the convective heating case. However, the opposite trend was attained in the convective cooling case. The contrast of LNN for different combinations of Rd and Hg (a–b) and Rd and Ri (c–d) with convective heating (a,c) and convective cooling (b,d) cases was presented in Figure 9a–d. It is seen from the graphical overview that the LNN grows when mounting the values of the radiation parameter and Richardson number, and it slumps when enhancing the heat consumption/generation parameter in the convective heating case. The reverse trend is obtained in the convective cooling case.

**Figure 10:**
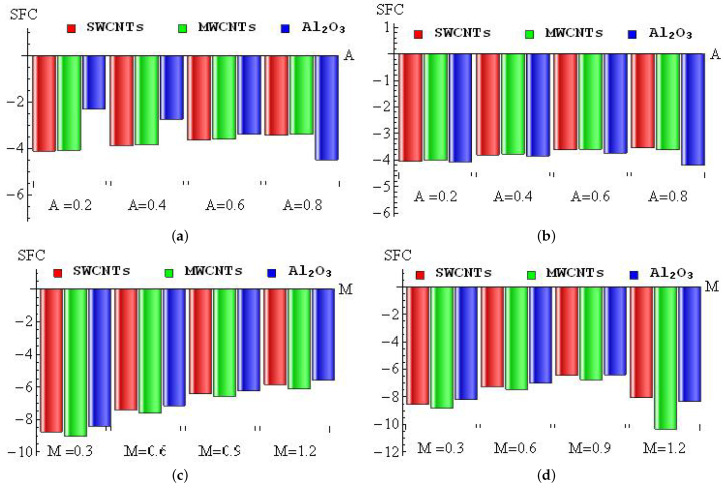
The diminishing percentage of SFC for *A* (**a**,**b**) and *M* (**c**,**d**) with convective heating (**a**,**c**) and convective cooling (**b**,**d**) cases.

The diminishing percentages of SFC for *A* (a–b) and *M* (c–d) with convective heating (a,c) and convective cooling (b,d) for SWCNTs, MWCNTs and $Al_{2}O_{3}$ nanofluid are drawn in Figure 10a–d. For the convective heating case, the maximum diminishing percentage (4.14%) occurred in $Al_{2}O_{3}$ nanofluid when changing *A* from 0 to 0.2, and the minimum diminishing percentage (3.41%) appeared in MWCNTs when changing *A* from 0.6 to 0.8, see Figure 10a. In the convective cooling case, the maximum diminishing percentage (4.21%) occurred in $Al_{2}O_{3}$ nanofluid when changing *A* from 0.6 to 0.8, and the minimum diminishing percentage (3.55%) appeared in SWCNTs when changing *A* from 0.6 to 0.8 (see Figure 10b). For the convective heating case, the maximum diminishing percentage (9.04%) occurred in MWCNTs when changing *M* from 0 to 0.3, and the minimum diminishing percentage (5.58%) appeared in $Al_{2}O_{3}$ nanofluid when changing *M* from 0.9 to 1.2 (see Figure 10c). In the convective cooling case, the maximum diminishing percentage (10.33%) occurred in MWCNTs when changing *M* from 0.9 to 1.2, and the minimum diminishing percentage (6.39%) appeared in $Al_{2}O_{3}$ nanofluid when changing *M* from 0.6 to 0.8 (see Figure 10d).

**Figure 11:**
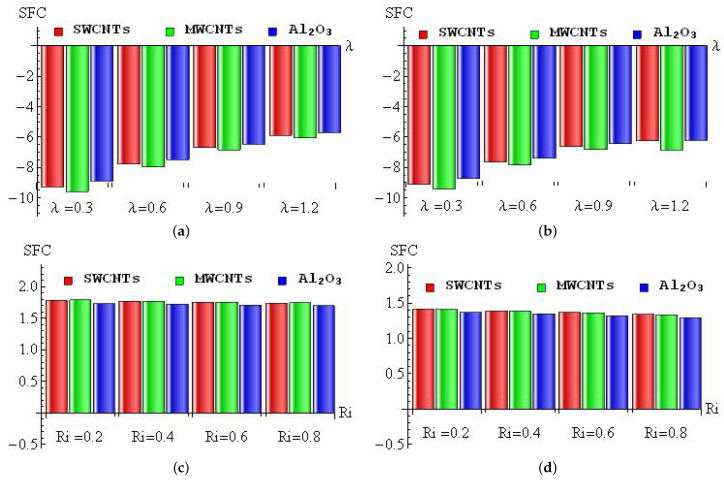
The diminishing/improving percentage of SFC for λ (**a**,**b**) and Ri (**c**,**d**) with convective heating (**a**,**c**) and convective cooling (**b**,**d**) cases.

Figure 11a–d is plotted to discuss the diminishing/improving percentage of SFC for λ (a–b) and Ri (c–d) with convective heating (a,c) and convective cooling (b,d) for SWCNTs, MWCNTs and $Al_{2}O_{3}$ nanofluid. For the convective heating case, the maximum diminishing percentage (9.58%) occurred in MWCNTs when changing λ from 0 to 0.3, and the minimum diminishing percentage (5.71%) appeared in $Al_{2}O_{3}$ nanofluid when changing λ from 0.9 to 1.2 (see Figure 11a). In the convective cooling case, the maximum diminishing percentage (9.43%) occurred in MWCNTs when changing λ from 0 to 0.3, and the minimum diminishing percentage (6.22%) appeared in $Al_{2}O_{3}$ nanofluid when changing λ from 0.9 to 1.2 (see Figure 11b). For the convective heating case, the maximum improving percentage (1.79%) occurred in MWCNTs when changing Ri from 0 to 0.2, and the minimum improving percentage (1.7%) appeared in $Al_{2}O_{3}$ nanofluid when changing Ri from 0.6 to 0.8 (see Figure 11c). In the convective cooling case, the maximum improving percentage (1.42%) occurred in SWCNTs when changing Ri from 0 to 0.2, and the minimum improving percentage (1.29%) appeared in $Al_{2}O_{3}$ nanofluid when changing Ri from 0.6 to 0.8 (see Figure 11d).

**Figure 12:**
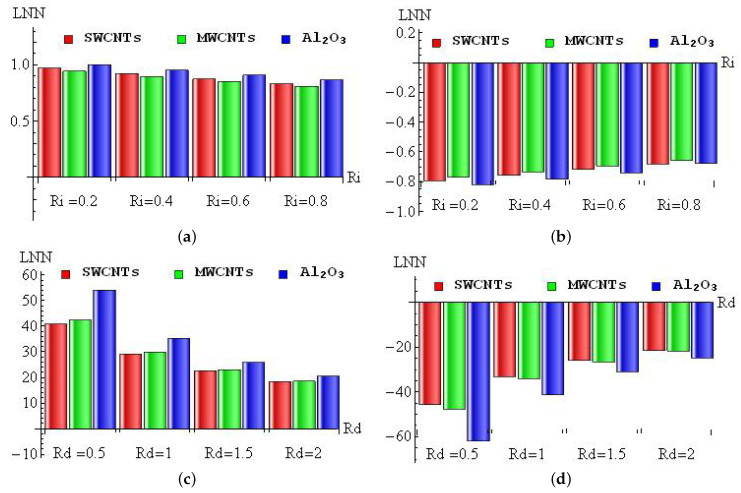
The diminishing/improving percentage of LNN for Ri (**a**,**b**) and Rd (**c**,**d**) with convective heating (**a**,**c**) and convective cooling (**b**,**d**) cases.

The diminishing/improving percentage of LNN for Ri (a–b) and Rd (c–d) with convective heating (a,c) and convective cooling (b,d) for SWCNTs, MWCNTs and $Al_{2}O_{3}$ nanofluid are sketched in Figure 12a–d. For the convective heating case, the maximum improving percentage (1%) occurred in $Al_{2}O_{3}$ nanofluid when changing Ri from 0 to 0.2, and the minimum improving percentage (0.8%) appeared in MWCNTs when changing Ri from 0.6 to 0.8 (see Figure 12a). In the convective cooling case, the maximum diminishing percentage (0.82%) occurred in $Al_{2}O_{3}$ nanofluid when changing Ri from 0 to 0.2, and the minimum diminishing percentage (0.66%) appeared in MWCNTs when changing Ri from 0.6 to 0.8 (see Figure 12b). For the convective heating case, the maximum improving percentage (54%) occurred in $Al_{2}O_{3}$ nanofluid when changing Rd from 0 to 0.5, and the minimum improving percentage (18.31%) appeared in SWCNTs when changing Rd from 1.5 to 2 (see Figure 12c). In the convective cooling case, the maximum diminishing percentage (61%) occurred in $Al_{2}O_{3}$ nanofluid when changing Rd from 0 to 0.5, and the minimum diminishing percentage (21.43%) appeared in SWCNTs when changing Rd from 1.5 to 2 (see Figure 12d).

**Figure 13:**
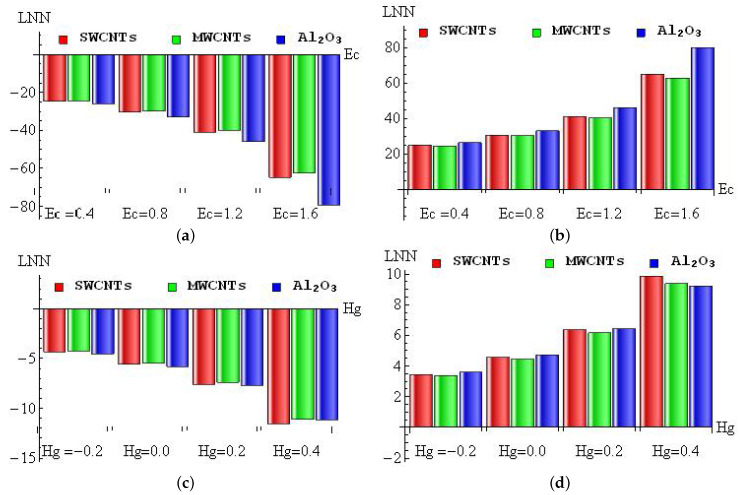
The diminishing/improving percentage of LNN for Ec (**a**,**b**) and Hg (**c**,**d**) with convective heating (**a**,**c**) and convective cooling (**b**,**d**) cases.

Figure 13a–d is taken to examine the diminishing/improving percentage of LNN for Ec (a–b) and Hg (c–d) with convective heating (a,c) and convective cooling (b,d) for SWCNTs, MWCNTs and $Al_{2}O_{3}$ nanofluid. For the convective heating case, the maximum diminishing percentage (79%) occurred in $Al_{2}O_{3}$ nanofluid when changing Ec from 1.2 to 1.6, and the minimum diminishing percentage (24.28%) appeared in MWCNTs when changing Ec from 0 to 0.4 (see Figure 13a). In the convective cooling case, the maximum improving percentage (79.83%) occurred in $Al_{2}O_{3}$ nanofluid when changing Ec from 1.2 to 1.6, and the minimum improving percentage (24.54%) appeared in MWCNTs when changing Ec from 0 to 0.4 (see Figure 13b). For the convective heating case, the maximum diminishing percentage (11.57%) occurred in SWCNTs when changing Hg from 0.2 to 0.4, and the minimum diminishing percentage (4.25%) appeared in MWCNTs when changing Hg from −0.4 to 0.2 (see Figure 13c). In the convective cooling case, the maximum improving percentage (9.85%) occurred in SWCNTs when changing Hg from 0.2 to 0.4, and the minimum improving percentage (3.34%) appeared in MWCNTs when changing Hg from −0.4 to 0.2 (see Figure 13d).

## 5. Conclusions

The impact of the thermally radiative MHD flow of CNTs/$Al_{2}O_{3}$ nanofluid in water past a stretchy plate embedded in a porous medium with the availability of heat consumption/generation and injection/suction was studied. The two varieties of CNTs, such as single-wall carbon nanotubes (SWCNTs) and multi-wall carbon nanotubes (MWCNTs), were taken into account. The amended expressions and their correlated constraints were numerically and analytically computed via MATLAB BVP4C and HAM theory, respectively. The primary outcomes of our investigation are as follows:

- Larger magnetic field and porosity parameters lead to declines in the fluid velocity.
- The fluid temperature is strengthened in opposition to the larger Biot number, Eckert number and radiation parameter.
- Decay in surface drag force is noted against a larger magnetic field and unsteady parameters.
- The radiation parameter leads to an improvement in the heat transfer gradient in the convective heating case, while it decays in the convective cooling case.
- The MWCNTs have higher skin friction values compared to SWCNTs and $Al_{2}O_{3}$ nanofluid.
- The lower heat transfer gradient appears in SWCNTs compared to MWCNTs and $Al_{2}O_{3}$ nanofluid.

## Figures and Tables

**Table 1:** Physical properties.

Physical Characteristics	SWCNTs	MWCNTs	${\mathrm{Al}}_{2}O_{3}$	Water
*k*	6600	3000	40	0.613
ρ	2600	1600	3970	997.1
cp	425	796	765	4179

**Table 2:** HAM order and numerical value of SWCNTs.

Order	$-F^{\prime \prime }\left(0\right)$		$-\Theta^{\prime }\left(0\right)$	
	HAM	NM	HAM	NM
1	1.33342		0.17483	
5	1.38956		0.15055	
10	1.39203		0.14900	
15	1.39203	1.39203	0.14882	0.14879
18	1.39203		0.14880	
20	1.39203		0.14880	
25	1.39203		0.14880	

**Table 3:** HAM order and numerical value of MWCNTs.

Order	$-F^{\prime \prime }\left(0\right)$		$-\Theta^{\prime }\left(0\right)$	
	HAM	NM	HAM	NM
1	1.32570		0.18125	
5	1.36868		0.15715	
10	1.37110		0.15554	
15	1.37110	1.37110	0.15534	0.15531
18	1.37110		0.15534	
20	1.37110		0.15534	
25	1.37110		0.15534	

**Table 4:** HAM order and numerical value of $Al_{2}O_{3}$ nanofluid.

Order	$-F^{\prime \prime }\left(0\right)$		$-\Theta^{\prime }\left(0\right)$	
	HAM	NM	HAM	NM
1	1.33487		0.22682	
5	1.41937		0.19447	
10	1.42201		0.19110	
15	1.42208	1.42210	0.19056	0.19044
18	1.42210		0.19046	
20	1.42210		0.19046	
25	1.42210		0.19046	

**Table 5:** The SFC for various values of *A*, *M*, λ, Ri, fw, Rd, Ec and Hg.

								SFC		
A	M	λ	Ri	fw	Rd	Ec	Hg	SWCNTs	MWCNTs	${\mathrm{Al}}_{2}O_{3}$
0	0.3	0.3	0.5	0.3	0.4	0.5	−0.4	−1.48053	−1.45882	−1.51221
0.3								−1.57165	−1.54773	−1.60573
0.5								−1.63077	−1.60542	−1.66642
0.8								−1.71685	−1.68943	−1.75482
1								−1.77244	−1.74371	−1.81193
0.2	0	0.3	0.5	0.3	0.4	0.5	−0.4	−1.41740	−1.39248	−1.45261
	0.3							−1.54159	−1.51841	−1.57488
	0.6							−1.65582	−1.63397	−1.68758
	0.9							−1.76229	−1.74156	−1.79277
	1.2							−1.86551	−1.84829	−1.89280
0.2	0.3	0	0.5	0.3	0.4	0.5	−0.4	−1.41056	−1.38556	−1.44619
		0.3						−1.54159	−1.51841	−1.57488
		0.6						−1.66138	−1.63957	−1.69284
		0.9						−1.77247	−1.75176	−1.80246
		1.2						−1.87707	−1.8578	−1.90547
0.2	0.3	0.3	0	0.3	0.4	0.5	−0.4	−1.6122	−1.58809	−1.64490
			0.3					−1.56924	−1.54571	−1.60236
			0.5					−1.54159	−1.51841	−1.57488
			0.8					−1.50147	−1.47875	−1.53487
			1					−1.47556	−1.45312	−1.50897
0.2	0.3	0.3	0.5	−0.8	0.4	0.5	−0.4	−1.01101	−1.00433	−1.01583
				−0.4				−1.17455	−1.16352	−1.18715
				0				−1.37146	−1.35429	−1.39482
				0.4				−1.60245	−1.57698	−1.63935
				0.8				−1.86531	−1.82942	−1.91800
0.2	0.3	0.3	0.5	0.3	0	0.5	−0.4	−1.54375	−1.52064	−1.57768
					0.5			−1.54107	−1.51787	−1.57420
					1			−1.53859	−1.5153	−1.57098
					1.5			−1.53625	−1.51287	−1.56795
					2			−1.53402	−1.51056	−1.56508
0.2	0.3	0.3	0.5	0.3	0.4	0	−0.4	−1.60216	−1.57806	−1.63474
						0.4		−1.55337	−1.53001	−1.58655
						0.8		−1.50719	−1.48448	−1.54070
						1.2		−1.46335	−1.44119	−1.49699
						1.6		−1.42158	−1.39990	−1.45524
0.2	0.3	0.3	0.5	0.3	0.4	0.5	−0.5	−1.54475	−1.5215	−1.57787
							−0.3	−1.53808	−1.51497	−1.57158
							0	−1.52464	−1.50188	−1.55926
							0.3	−1.50332	−1.48148	−1.54099
							0.5	−1.47374	−1.45395	−1.51759

**Table 6:** The LNN for various values of *A*, *M*, λ, Ri, fw, Rd, Ec and Hg.

								LNN		
A	M	λ	Ri	fw	Rd	Ec	Hg	SWCNTs	MWCNTs	${\mathrm{Al}}_{2}O_{3}$
0	0.3	0.3	0.5	0.3	0.4	0.5	−0.4	0.33002	0.33444	0.34248
0.3								0.33686	0.34135	0.34981
0.5								0.34076	0.34530	0.35399
0.8								0.34579	0.35043	0.35942
1								0.34871	0.35340	0.36257
0.2	0	0.3	0.5	0.3	0.4	0.5	−0.4	0.36994	0.37517	0.38588
	0.3							0.33473	0.33919	0.34752
	0.6							0.30204	0.30583	0.31181
	0.9							0.27131	0.27450	0.27816
	1.2							0.24136	0.24339	0.24598
0.2	0.3	0	0.5	0.3	0.4	0.5	−0.4	0.34947	0.35424	0.36373
		0.3						0.33473	0.33919	0.34752
		0.6						0.32082	0.32500	0.33222
		0.9						0.30758	0.31150	0.31764
		1.2						0.29488	0.29855	0.30366
0.2	0.3	0.3	0	0.3	0.4	0.5	−0.4	0.32704	0.33161	0.33929
			0.3					0.33175	0.33625	0.34433
			0.5					0.33473	0.33919	0.34752
			0.8					0.33897	0.34337	0.35210
			1					0.34165	0.34603	0.35502
0.2	0.3	0.3	0.5	−0.8	0.4	0.5	−0.4	0.32262	0.32433	0.32920
				−0.4				0.32552	0.32813	0.33391
				0				0.330157	0.33381	0.34091
				0.4				0.33638	0.34110	0.34987
				0.8				0.34309	0.34875	0.35920
0.2	0.3	0.3	0.5	0.3	0	0.5	−0.4	0.25220	0.25333	0.24256
					0.5			0.35542	0.36071	0.37386
					1			0.45898	0.46836	0.50551
					1.5			0.56237	0.57575	0.63658
					2			0.66535	0.68263	0.76666
0.2	0.3	0.3	0.5	0.3	0.4	0	−0.4	0.48135	0.48518	0.51258
						0.4		0.36302	0.36737	0.37943
						0.8		0.25269	0.25745	0.25491
						1.2		0.14947	0.15457	0.13820
						1.6		0.05260	0.05798	0.02854
0.2	0.3	0.3	0.5	0.3	0.4	0.5	−0.5	0.34101	0.34545	0.35443
							−0.3	0.32784	0.33233	0.33999
							0	0.30233	0.30698	0.31247
							0.3	0.26491	0.27013	0.27372
							0.5	0.22272	0.22947	0.23333

## Nomenclature

Symbols	Description
a	positive constant
α	thermal diffusivity
B	applied magnetic field
$B_{0}$	intensity of the magnetic field
$C_{F}$	skin friction coefficient
$c_{p}$	capacity of specific heat
$h_{c}$	heat transfer coefficient
$k_{1}{}^{*}$	thermal conductivity
$k^{*}$	the mean absorption coefficient
nf,f	subscript represents nanofluid and base fluid
ν	kinematic viscosity
Υ	dimensionless variable
Q	heat generation or absorption coefficient
$Q_{0}$	heat generation or absorption constant
ρ	density
$\overset{˘}{T}$	non-dimensional temperature
${\overset{˘}{T}}_{f}$	temperature of the hot fluid
${\overset{˘}{T}}_{w}$	surface temperature
${\overset{˘}{T}}_{\infty }$	ambient temperature
Θ	dimensionless temperature
$\overset{˘}{u},\overset{˘}{v}$	velocity components
$\overset{˘}{x},\overset{˘}{y}$	Cartesian coordinates
$A\left(=\frac{\xi }{a}\right)$	unsteady parameter
$Bi\left(=\frac{h_{c}}{k_{f}}\sqrt{\frac{\nu_{f}\left(1-\xi t\right)}{a}}\right)$	Biot number
$Ec\left(=\frac{U_{w}^{2}}{{\left(cp\right)}_{f}\left({\overset{˘}{T}}_{f}-{\overset{˘}{T}}_{\infty }\right)}\right)$	Eckert number
$fw\left(=\frac{v_{0}}{\sqrt{a\nu_{f}}}\right)$	suction/injection parameter
$Hg\left(=\frac{Q_{0}\sqrt{\left(1-\xi t\right)}}{a{\left(\rho cp\right)}_{f}}\right)$	heat consumption/generation
$\lambda \left(=\frac{\nu_{f}}{k_{1}^{*}a\left(1-\xi t\right)}\right)$	porosity parameter
$M\left(=\frac{\sigma_{f}B_{0}^{2}}{a\rho_{f}}\right)$	magnetic field parameter
$Pr\left(=\frac{\nu_{f}}{\alpha_{f}}\right)$	Prandtl number
$Re\left(=\sqrt{\frac{a{\overset{˘}{x}}^{2}}{\left(1-\xi \overset{˘}{t}\right)\nu_{f}}}\right)$	Reynolds number
$Ri\left(=\frac{g\beta_{f}\left({\overset{˘}{T}}_{f}-{\overset{˘}{T}}_{\infty }\right)}{U_{w}^{2}}\right)$	Richardson number
$Rd\left(=\frac{4\sigma^{*}{\overset{˘}{T}}_{\infty }^{3}}{k^{*}k_{f}}\right)$	radiation parameter

## Abbreviations

CCHF	Cattaneo-Christov heat flux
CNTs	carbon nanotubes
HAM	homotopy analysis method
LNN	local Nusselt number
MHD	magnetohydrodynamics
MWCNTs	multi-wall carbon nanotubes
NPVF	nanoparticle volume fraction
ODE	ordinary differential equations
PDE	partial differential equations
SFC	skin friction coefficient
SS	stretching sheet
SWCNTs	single wall carbon nanotubes

## Appendix A

The thermophysical properties are mathematically defined as

$$\begin{array}{ccc} & & A_{1}=\frac{\mu_{nf}}{\mu_{f}}=\frac{1}{{\left(1-\phi \right)}^{2.5}};\\ & & A_{2}=\frac{\rho_{f}}{\rho_{nf}}=\frac{1}{\left(1-\phi +\phi \frac{\rho_{CNT}}{\rho_{f}}\right)};\\ & & A_{3}=\frac{\sigma_{nf}}{\sigma_{f}}=1+\frac{3\left(\frac{\sigma_{CNT}}{\sigma_{f}}-1\right)\phi }{\left(\frac{\sigma_{CNT}}{\sigma_{f}}+2\right)-\phi \left(\frac{\sigma_{CNT}}{\sigma_{f}}-1\right)};\\ & & A_{4}=\frac{{\left(\rho \beta \right)}_{nf}}{{\left(\rho \beta \right)}_{f}}=1-\phi +\phi \frac{{\left(\rho \beta \right)}_{CNT}}{{\left(\rho \beta \right)}_{f}};\\ & & A_{5}=\frac{k_{nf}}{k_{f}}=\frac{\left(1-\phi \right)+2\phi \frac{k_{CNT}}{k_{CNT}-k_{f}}ln\frac{k_{CNT}+k_{f}}{2k_{f}}}{\left(1-\phi \right)+2\phi \frac{k_{f}}{k_{CNT}-k_{f}}ln\frac{k_{CNT}+k_{f}}{2k_{f}}};\\ & & A_{6}=\frac{{\left(\rho cp\right)}_{f}}{{\left(\rho cp\right)}_{nf}}=\frac{1}{1-\phi +\phi \frac{{\left(\rho c_{p}\right)}_{CNT}}{{\left(\rho c_{p}\right)}_{f}}};\;\; \end{array}$$
